# Rapid Detection and Quantification of *Mycobacterium tuberculosis* DNA in Paraffinized Samples by Droplet Digital PCR: A Preliminary Study

**DOI:** 10.3389/fmicb.2021.727774

**Published:** 2021-09-13

**Authors:** Maria Antonello, Rossana Scutari, Calogero Lauricella, Silvia Renica, Valentina Motta, Stefania Torri, Cristina Russo, Leonarda Gentile, Valeria Cento, Luna Colagrossi, Giordana Mattana, Luigi Ruffo Codecasa, Chiara Vismara, Francesco Scaglione, Silvio Marco Veronese, Emanuela Bonoldi, Alessandra Bandera, Andrea Gori, Ester Mazzola, Carlo Federico Perno, Claudia Alteri

**Affiliations:** ^1^Department of Oncology and Hemato-Oncology, University of Milan, Milan, Italy; ^2^Department of Experimental Medicine, University of Rome “Tor Vergata,”Rome, Italy; ^3^Department of Pathology, ASST Grande Ospedale Metropolitano Niguarda, Milan, Italy; ^4^Unit of Microbiology, Department of Chemical-Clinical and Microbiology Analyses, ASST Grande Ospedale Metropolitano Niguarda, Milan, Italy; ^5^Unit of Microbiology and Diagnostic Immunology, Bambino Gesù Children’s Hospital, IRCCS, Rome, Italy; ^6^Regional TB Reference Centre, Villa Marelli Institute, ASST Grande Ospedale Metropolitano Niguarda, Milan, Italy; ^7^Infectious Disease Unit, Department of Internal Medicine, Fondazione IRCCS Ca’ Granda, Ospedale Maggiore Policlinico, Milan, Italy; ^8^Department of Pathophysiology and Transplantation, University of Milan, Milan, Italy; ^9^Multimodal Medicine Research Area, Bambino Gesù Children’s Hospital, IRCCS, Rome, Italy

**Keywords:** MTB, ddPCR, MTB diagnosis, *tuberculosis*, extrapulmonary TB

## Abstract

**Background:** Rapid and reliable diagnosis of tuberculosis (TB) represents a diagnostic challenge in compartmentalized extrapulmonary TB infection because of the small number of mycobacteria (MTB) and the frequent lack of fresh samples to perform culture. Here, we estimate the performances of homemade droplet digital PCR (ddPCR)-based assays against culture in 89 biopsies, for those fresh and formalin-fixed and paraffin-embedded (FFPE) subsamples were available.

**Methods:** MTB diagnosis in fresh subsamples was performed by culture. Fresh subsamples were also analyzed for acid-fast bacilli smear-microscopy (AFB) and Xpert^®^ MTB/RIF (Xpert). MTB examination was repeated in blind in the 89 FFPE subsamples by in-house ddPCR assays targeting the IS6110 and rpoB. Analytical sensitivity of ddPCR assays was evaluated using serial dilution of H37Rv strain. Limit of detection (LOD) was calculated by probit analysis. Results were expressed in copies/10^6^ cells.

**Results:** IS6110 and rpoB ddPCR assays showed a good linear correlation between expected and observed values (*R*^2^: 0.9907 and 0.9743, respectively). Probit analyses predicted a LOD of 17 and 40 copies/10^6^ cells of MTB DNA for IS6110 and rpoB, respectively. Of the 89 biopsies, 68 were culture positive and 21 were culture negative. Considering mycobacterial culture as reference method, IS6110 assay yielded positive results in 67/68 culture-positive samples with a median interquartile range (IQR) of 1,680 (550–8,444) copies/10^6^ cells (sensitivity: 98.5%; accuracy: 98.9). These performances were superior to those reported by the rpoB assay in FFPE subsamples (sensitivity: 66.20%; accuracy: 74.1) and even superior to those reported by Xpert and AFB in fresh subsamples (sensitivity: 79.4 and 33.8%, respectively; accuracy: 84.3 and 49.4, respectively). When Xpert and AFB results were stratified according to mycobacterial load detected by rpoB and IS6110 ddPCR, bacterial load was lower in Xpert and AFB negative with respect to Xpert and AFB-positive samples (*p* = 0.003 and 0.01 for rpoB and *p* = 0.01 and 0.11 for IS6110), confirming the poor sensitivity of these methods in paucibacillary disease.

**Conclusion:** ddPCR provides highly sensitive, accurate, and rapid MTB diagnosis in FFPE samples, as defined by the high concordance between IS6110 assay and culture results. This approach can be safely introduced in clinical routine to accelerate MTB diagnosis mainly when culture results remain unavailable.

## Introduction

Tuberculosis (TB) is a multisystemic disease caused by *Mycobacterium tuberculosis* complex (MTB). The pathogen primarily infects the lungs (pulmonary TB) but can also affect other structural parts of the human anatomy (extrapulmonary TB), such as lymph nodes, intestines, pleura, skin, and bones (Noussair et al., 2009).

It affects approximately 2 billion people worldwide, especially in developing countries, and stands as the leading cause of death from a single infectious agent [World Health Organization (WHO), 2012].

To reduce the mortality of MTB infection, to prevent its spread, and to start the correct treatment, an early, accurate, and rapid diagnosis is essential.

To date, the conventional approach for MTB diagnosis is mainly based on microscopic detection of acid-fast bacilli in smears (AFB), followed by MTB culture (Ryu, 2015; Caulfield and Wengenack, 2016). While AFB is a laborious method characterized by variable sensitivity (71.4% in lung samples and 24% in extrapulmonary samples) (Karadaǧ et al., 2013), mycobacterium culture is considered the gold standard method for MTB diagnosis as it has high specificity but requires a long time of incubation, up to 8 weeks for a certain negativity (Forbes et al., 2018).

For these reasons, in the case of extrapulmonary MTB infection, the diagnosis is challenging because of the small amount of MTB present at the sites of the disease and the difficulty of obtaining culture results (Golden, 2005; Noussair et al., 2009). In the case of surgically resected tissues fixed in formalin, mycobacterial culture is not feasible at all, and a correct diagnosis based on pathological features and AFB smear is difficult. Acid-fast staining for MTB on formalin-fixed and paraffin-embedded (FFPE) tissue has indeed a very low sensitivity, ranging between 3 and 60% (Fukunaga et al., 2002; Ahmed et al., 2011; Eshete et al., 2011), and some histological findings, like granuloma and necrosis, typically found in many other diseases including sarcoidosis and fungal infections (El-Zammar and Katzenstein, 2007), make MTB diagnosis particularly challenging.

In the last decade, real-time (RT) PCR assays have been introduced in laboratory routine, thanks to their sensitivity and their shortened turnaround time (Forbes and Hicks, 1993; Marchetti et al., 1998; Moure et al., 2019). Most of these methods are based on the detection of multi-copy insertion sequences (IS, such as IS986, IS987, IS1081, and IS6110), which should increase the sensitivity of the assays (Bisognin et al., 2018; Lin et al., 2021). Among these RT-PCR-based assays, Xpert^®^ MTB/RIF (Cepheid, Sunnyvale, CA, United States) (Xpert) is recommended by the World Health Organization (WHO) as rapid molecular diagnostic test for adults and children also in the case of extrapulmonary and FFPE specimens [Lee et al., 2011; Mazzola et al., 2016; Yang et al., 2017; Moure et al., 2019; Nyaruaba et al., 2019; World Health Organization (WHO), 2020], although it has a low diagnostic accuracy in paucibacillary disease (Allahyartorkaman et al., 2019).

Among other molecular platforms, droplet digital PCR (ddPCR) is a highly sensitive method widely used for the detection of a variety of pathogens, thanks to its ability to reliably detect down to a few copies of genomes (Caviglia et al., 2018; Alteri et al., 2020; Chen et al., 2021; Malin et al., 2021). Currently, two reports suggested that homemade ddPCR assays might provide a valid alternative for detecting MTB in extrapulmonary and/or FFPE samples (Yang et al., 2017; Cao et al., 2020), but as far as we know no study has defined the concordance between these molecular assays and the reference culture method.

Here, to evaluate the ddPCR-based method as suitable alternative for routine clinical diagnoses of MTB in FFPE samples, the performances of two MTB ddPCR-based assays were compared with the gold-standard culture methods and with the conventional AFB and Xpert in a set of tissues, for those fresh and FFPE subsamples were available.

## Materials and Methods

### Clinical Sample Collection

A total of 89 consecutive biopsies from different anatomical districts of patients with a clinical suspect of TB were retrospectively collected at ASST Grande Ospedale Metropolitano Niguarda (Milan, Italy) between 2013 and 2019. Samples were selected according to clinical suspect of MTB based on radiology findings, cytology reports, and/or medical history of patients including previous MTB treatment [World Health Organization (WHO), 2013]. The distribution of samples against year of collection is reported in Supplementary Table 1. Biopsies were subdivided in two subsamples by trained medical personnel. One fresh subsample was immediately tested for MTB diagnosis by culture methods, using both solid (Lowenstein–Jensen) and liquid (MGIT 960; Becton Dickinson Biosciences, Sparks, MD, United States) media [European Centre for Disease Prevention and Control (ECDC), 2018; Riccardi et al., 2020]. Fresh subsamples were also analyzed for AFB microscopy and Xpert^®^ MTB/RIF (Cepheid, Sunnyvale, CA, United States) (Tortoli et al., 2012). As required by WHO and ECDC guidelines [World Health Organization (WHO), 2012; European Centre for Disease Prevention and Control (ECDC), 2018], all these procedures took place in a Biosafety Level 3 (BSL3) laboratory, with limited access, using required personal protective equipment and following control measures and all procedures to minimize aerosol and droplet formation. The residual sample was stored as formalin fixed and paraffin embedded (FFPE) (Slaoui and Fiette, 2011; Sadeghipour and Babaheidarian, 2019) for alternative diagnosis by histopathological examination and archived in a biobank for later use.

The study was conducted in accordance with the principles of the 1964 Declaration of Helsinki. The related information of the samples was processed by maintaining anonymization measures. Due to the non-interventional nature of the study and according to the applicable relevant national legislation and local rules, approval of the local Research Ethics Committee and informed consent were not mandatory.

### DNA Extraction and *Mycobacteria tuberculosis* Detection in Formalin-Fixed and Paraffin-Embedded Samples

MTB detection was repeated in blind in the stored FFPE samples by using ddPCR homemade assays. In brief, after sample deparaffinization (Fu et al., 2016), total DNA was extracted using Maxwell CSC DNA FFPE kit (Promega, Madison, WI, United States) following the instruction of the manufacturer (Sarnecka et al., 2019).

MTB DNA was quantified by means of the QX200^TM^ Droplet Digital PCR System (Biorad) using homemade assays targeting the multicopy gene *IS6110* (Forward: 5′-ATCTGGAC CCGCCAA-3′; Reverse: 5′-CCTATCCGTATGGTGGATAA-3′, and HEX Probe: 5′-AGGTCGAGTACGCCTT-3′) and the single-copy gene *rpoB* (Forward: 5′-GGAGCGCCAAACCG-3′; Reverse: 5′-AGTCCCGGAACCTCAA-3′, and FAM Probe: 5′-TTCGCTAAGCTGCGC-3′). The human albumin was used as housekeeping gene (ddPCR^TM^ Copy Number Assay: ALB, Human, dHsaCNS864404398).

The cycling condition was the following: 95°C (10 min), 39 cycles of 94°C (30 s), and 56°C (1 min), 98°C (10 min), 4°C (∞). A sample was considered “positive and quantifiable” if at least two droplets (in *IS6110* or *rpoB* assay) were observed.

MTB DNA (copies/reaction) was then normalized into number copies/10^6^ cells. In detail, raw data obtained were converted into copies/10^6^ cells according to the following formula: [MTB-DNA (copies/10^6^ cells) = MTB-DNA raw data × 10^6^ cells/(housekeeping gene copies/2)].

### Accuracy and Limit of Detection of Droplet Digital PCR Assay

To verify the correct performance of MTB detection and quantification, the laboratory-cultured H37Rv strain (previously inactivated by incubation at 95°C for 20 min and sonication at room temperature for 15 min) served as quantitation standards in two independent runs. Five serial dilutions were prepared in order to deposit 10^4^, 10^3^, 10^2^, 10, and 1 copy(ies) of the MTB genome (1 ng = 168,100 copies) in 3 μg of human genomic DNA, corresponding to 10^6^ cells. The first three dilutions were repeated in duplicate, while the remaining ones were in triplicate. To determine the specificity and cross-reactivity of the MTB ddPCR-based assays, the DNA of cultured non-tuberculous strains [*M. abscessus* subsp. *abscessus* (MBABAB) and *M. abscessus* subsp. *bolletii* (MBABBO)], and negative controls (*n* = 20) were also added in each run. The negative controls belonged to FFPE samples from subjects without clinical and bacteriological signs of MTB infection [World Health Organization (WHO), 2013].

Coefficient of determination (*R*^2^) of MTB quantification was assessed for both *IS6110* and *rpoB* by linear regression analysis by plotting the measured copies of the standards and comparing them with expected values of serial dilutions. The coefficient of variation (CV) was calculated as the standard deviation (SD) of copies/10^6^ cells divided by replicate mean.

The lower limit of blank (LoB) was determined by testing the replicates of the 20 negative controls, according to the following formula: LoB = mean of blank + 1.645 × (SD of blank) (Armbruster and Pry, 2008). The limit of detection (LoD) was determined by probit regression analysis.

### Statistical Analyses

Sensitivity and specificity of ddPCR, Xpert, and AFB in terms of the ability to correctly diagnose MTB in tissue samples (pulmonary and extrapulmonary) were evaluated against culture results.

Reproducibility of quantification methods was assessed by intra- and inter-run tests using serial standard dilutions, and the differences between the expected and observed values were expressed as the mean ± SD *IS6110* and *rpoB* copy numbers.

Descriptive statistics were expressed as median values and interquartile range (IQR) for continuous variables and absolute number and frequency (percentage) for categorical variables. To assess significant differences, Fisher exact or Kruskal–Wallis test and Wilcoxon rank sum test were used for categorical and continuous variables, respectively. A *p*-value < 0.05 was considered statistically significant. All statistical analyses were performed with SPSS software package for Windows (version 25.0, SPSS INC., Chicago, IL, United States).

## Results

### Study Population

The demographic and clinical characteristics of the population included in the study are reported in Table 1. Samples were retrieved mainly by male (57.3%) with a median age of 36 years (IQR: 27–53). As expected, extrapulmonary samples were the majority (85, 95.5%) and mainly from lymphatic system (52, 58.4%) followed by pleural samples (17, 19.1%). Sixty-eight samples (76.4%) were MTB positive because of the positive mycobacterial culture from the fresh sample [median (IQR) days for culture positivity: 11 (13–16)]. The remaining 21 samples were MTB negative because of the negative culture and subsequent different diagnosis. Most of the positive samples (94.1%) belonged to patients receiving their first TB diagnosis at the time of the biopsy, and only 5.9% belonged to patients with a previously MTB-positive known condition.

**TABLE 1:** Demographic and clinical data of the sampled population.

	Overall	Culture positive (*N* = 68)	Culture negative (*N* = 21)	*p*-Value^*a*^
Male, *n* (%)	51 (57.3)	41 (60.3)	10 (47.6)	0.325
**Age, median (IQR)**	36 (27–53)	35 (26–45)	54 (35–71)	**0.006**
<40, *n* (%)	54 (60.7)	47 (69.1)	7 (33.3)	**0.005**
≥40, *n* (%)	35 (39.3)	21 (30.9)	14 (66.7)	
**TB classes**				
New case of TB, *n* (%)	64 (71.9)	64 (94.1)	–	–
Case of previously diagnosticated TB, *n* (%)	4 (4.5)	4 (5.9)	–	–
**Viral coinfections, *n* (%)**	8 (9)	7 (10.3)	1 (4.8)	0.675
**HIV co-infection, *n* (%)**	4 (4.5)	4 (5.9)	0 (0.0)	0.569
**Anatomical districts**				
Pulmonary site, *n* (%)	4 (4.5)	1 (1.5)	3 (14.3)	**0.039**
Extrapulmonary site, *n* (%)	85 (95.5)	67 (98.5)	18 (85.7)	
Lymphatic system, *n* (%)	52 (58.4)	44 (64.7)	8 (38.1)	**0.043**
Pleura, *n* (%)	17 (19.1)	15 (22.0)	2 (9.5)	0.341
Musculoskeletal apparatus, *n* (%)	4 (4.5)	1 (1.5)	3 (14.3)	**0.039**
Gastrointestinal tract, *n* (%)	4 (4.5)	4 (5.9)	0 (0.0)	0.569
Others^*b*^, *n* (%)	8 (9.0)	3 (4.4)	5 (23.8)	**0.016**
**Previous MTB results^*c*^**				
Smear positive test, *n* (%)	23 (25.8)	23 (33.8)	0 (0.0)	–
Xpert MTB/RIF positive test, *n* (%)	54 (60.7)	54 (79.4)	0 (0.0)	–

*IQR, interquartile range; TB, tuberculosis; MTB, *Mycobacterium tuberculosis*.*

*^*a*^Fisher exact test and Wilcoxon rank sum test were used for categorical and continuous variables, respectively. Statistically significant differences (*p*-values < 0.05) are highlighted in bold.*

*^*b*^Soft tissue biopsy (*n* = 4); hearth biopsy (*n* = 2); spinal cord biopsy (*n* = 1), nasal biopsy (*n* = 1).*

*^*c*^Performed on fresh subsamples.*

No differences were found between MTB-positive and -negative samples, with the sole exception of patients aged below 40 years, most frequently found among MTB-positive samples (Table 1). Viral coinfections were quite rare (9.0%) and prevalently found in patients with positive MTB culture (4.5%, no significant data).

### Performance of Droplet Digital PCR-Based Assays

#### Assay Linearity and Limit of Detection

**FIGURE 1:**
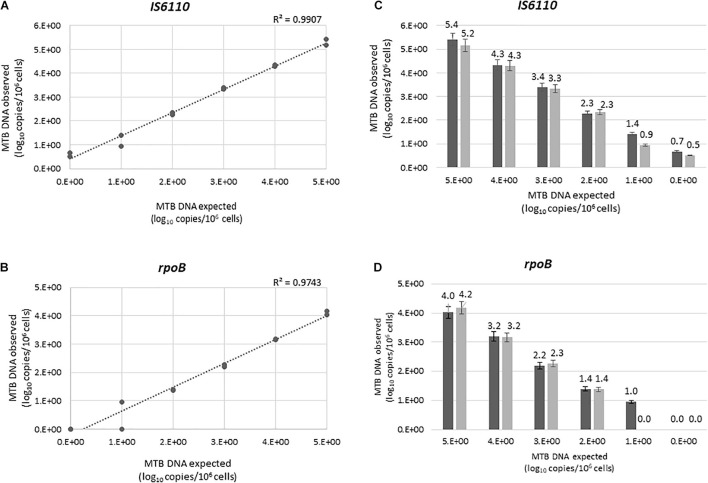
Linear correlations between the expected and the observed *IS6110* **(A)** and *rpoB* **(B)** load, expressed as log_10_ copies/10^6^ cells. *IS6110* **(C)** rpoB **(D)** load in the first and second experiment were shown in dark and light gray, respectively. Each value was tested in two independent experiments, each led in duplicate. Bars represent mean (+SD).

The linearity of the ddPCR assays was tested by quantifying serial dilutions of a known amount of MTB DNA. Our method showed a good linear correlation between expected and observed MTB DNA quantification, for both *IS6110* (*R*^2^ = 0.9907) and *rpoB* (*R*^2^ = 0.9743) (Figures 1A,B). No signal was detected in any of the 20 certainly negative samples tested, nor in the non-tuberculous extract (MBABAB and MBABBO) added in each run (Supplementary Figure 1). An example of Quantasoft panel for *IS6110* and *rpoB* obtained by the positive MTB DNA control, four positive samples, and a negative sample are also reported in Supplementary Figure 2.

#### Intra-run and Inter-run Reproducibility

Intra-run reproducibility analysis confirmed the high reliability of the methods (Figures 1C,D). The mean (±SD) differences between the expected and observed MTB copy number per 10^6^ cells (expressed as log_10_) were for *IS6110* assay: −0.363 ± 0.07 for 10^5^, −0.338 ± 0.02 for 10^4^, −0.338 ± 0.05 for 10^3^, −0.340 ± 0.06 for 10^2^, −0.338 ± 0.12 for 10 copies, −0.348 ± 0.04 for one copy; for *rpoB* assay: 0.898 ± 0.10 for 10^5^, 0.840 ± 0.05 for 10^4^, 0.802 ± 0.08 for 10^3^, 0.602 ± 0.02 for 10^2^, −0.060 ± 0.40 for 10 copies, −0.348 ± 0.04 for one copy. Mean CVs of the two experiments were 3.48 for *IS6110*, and 7.85 for *rpoB*.

The analysis of inter-run reproducibility confirmed the above results. The mean (±SD) differences between the expected and observed MTB copy number per 10^6^ cells (expressed as log_10_) were for *IS6110* assay: −0.292 ± 0.17 for 10^5^, −0.317 ± 0.03 for 10^4^, −0.367 ± 0.04 for 10^3^, −0.307 ± 0.05 for 10^2^, −0.176 ± 0.33 for 10, and −0.584 ± 0.1 for one copy; for *rpoB* assay: 0.899 ± 0.10 for 10^5^, 0.824 ± 0.02 for 10^4^, 0.764 ± 0.05 for 10^3^, 0.611 ± 0.01 for 10^2^, 0.523 ± 0.67 for 10, −0.090 ± 0.16 for one copy. Mean CV was 9.04 and 24.61 for *IS6110* and *rpoB*, respectively.

### Sensitivity and Specificity of Droplet Digital PCR-Based Assays Against Culture

All the DNA extracts obtained by the 89 FFPE samples were of high quality and quantity as confirmed by human albumin quantification [median (IQR): 1,760 (1,228–2,200) copies/μl; Supplementary Figure 3].

The overall sensitivity of the ddPCR assays was 98.5 (95.6–100.0) for *IS6110* and 66.2 (54.9–77.4) for *rpoB*. No false-positive results were highlighted among the 21 culture-negative FFPE samples (Table 2).

**TABLE 2:** Performances of homemade ddPCR assays, Xpert^®^ MTB/RIF molecular assay, and AFB against MTB culture.

Method	Sensitivity (%) (95% CI)	Specificity (%) (95% CI)	PPV (%) (95% CI)	NPV (%) (95% CI)	Accuracy
*IS6110* ddPCR assay^*a*^	98.5 (95.6–100.0)	100.0 (86.3–100.0)	100.0 (95.7–100.0)	95.5 (82.3–100.0)	98.9
*rpoB* ddPCR assay^*a*^	66.2 (54.9–77.4)	100.0 (82.8–100.0)	100.0 (92.0–100.0)	47.7 (32.9–62.5)	74.1
Xpert^®^ MTB/RIF molecular assay^*b*^	79.4 (69.8–89.0)	100.0 (83.2–100.0)	100.0 (93.5–100.0)	60.0 (43.77–76.23)	84.3
AFB^*b*^	33.8 (22.6–45.1)	100.0 (83.0–100.0)	100.0 (84.5–100.0)	31.8 (20.6–43.1)	49.4

*CI, confidence interval; NPV, negative predicted value; PPV, positive predicted value, AFB acid-fast bacilli microscopy.*

*^*a*^Performed on FFPE subsamples.*

*^*b*^Performed on fresh subsamples.*

Among the 68 positive mycobacterial cultures, ddPCR yielded 67/68 (98.5%) positive results for *IS6110* with a median (IQR) of 1,680 (550–8,444) copies/10^6^ cells, and 45/68 (66.2%) positive results for *rpoB* with a median (IQR) of 308 (99–1,419) copies/10^6^ cells. One sample was negative for both *rpoB* and *IS6110*, 45 samples were positive for both *IS6110* and *rpoB* (double positive), and 22 were positive only for *IS6110* (single positive). Double-positive with respect to single-positive samples were characterized by higher mycobacterial loads [*IS6110* copies/10^6^ cells, median (IQR): 3,578 (1,352–11,983) vs. 308 (99–1,419), *p* = 0.001], but not by a shorter time to positive culture [days, median (IQR): 13 (11–16) vs. 12 (10–15), *p* = 0.687].

### Sensitivity and Specificity of Xpert and Acid-Fast Bacilli Against Culture

**FIGURE 2:**
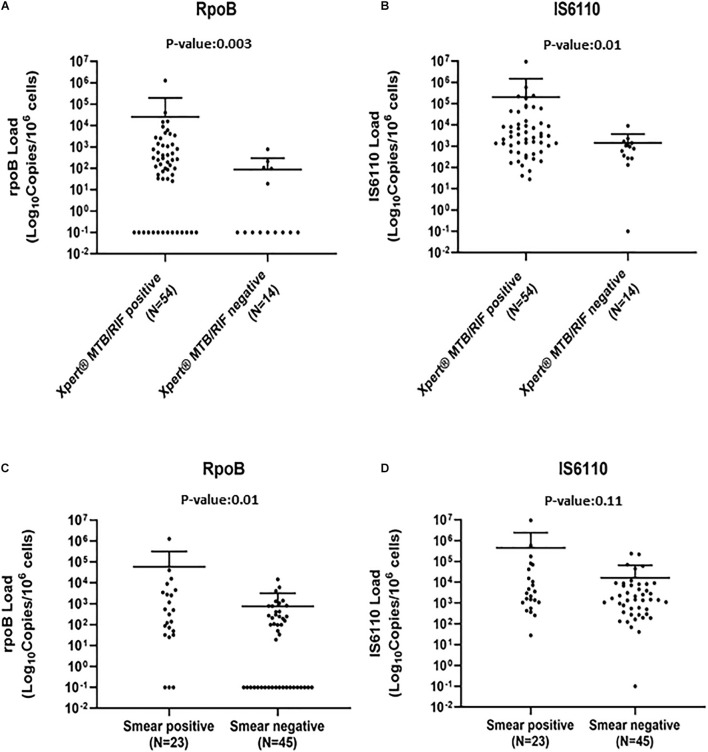
*Mycobacteria tuberculosis* (MTB) *rpoB* and *IS6110* load against Xpert **(A,B)** and acid-fast bacilli (AFB) smear **(C,D)** results. Each value was represented by a dot; bars represent median and interquartile range (IQR). *p*-Values were calculated by Wilcoxon rank sum test and Kruskal–Wallis where necessary.

When used in fresh subsamples, Xpert and AFB had a sensitivity of 79.4% (69.8–89.0) and 33.8% (22.6–45.1), respectively, lower than that observed with ddPCR in FFPE subsamples. As for ddPCR, no false-positive results were highlighted (Table 2). To define if the bacterial load can influence the sensitivity of Xpert and AFB, qPCR and smear results were stratified according to *rpoB* and *IS6110* load detected by ddPCR-assays. As expected, *rpoB* and *IS6110* loads were significantly lower in Xpert-negative with respect to Xpert-positive samples [*rpoB* load, median (IQR): 107 (97–210) vs. 414 (110–2,651), *p* = 0.003; *IS6110* load, median (IQR): 941 (361–1,412) vs. 2,892 (552–10,709), *p* = 0.01, Figures 2A,B]. Superimposable data were found for smear results (Figures 2C,D). No significant differences in the *IS6110* or *rpoB* loads were found against time of the first MTB diagnosis, anatomical districts, age, or sex of patients (Supplementary Figures 4A–H).

## Discussion

Results of this preliminary study clearly revealed that the ddPCR-based assay was non-inferior to the reference culture method for the detection of MTB in tissue biopsies, even when FFPE samples were considered. Moreover, the performances of *IS6110* ddPCR assay in detecting MTB in FFPE subsamples were superior to the standard Xpert^®^ MTB/RIF and AFB microscopy used for tuberculosis case detection in fresh subsamples, as well as defined by the sensitivities obtained in the 89 in-blind analyzed biopsies (sensitivity: 98.5 vs. 79.4 vs. 33.8%, respectively).

Due to the low sensitivity and specificity of AFB microscopy when compared with culture method (Karadaǧ et al., 2013; Molicotti et al., 2014; Pk et al., 2017; Bahr et al., 2018), molecular methods (as Xpert^®^ MTB/RIF assay) were introduced to improve the speed and specificity of TB diagnosis mainly in the context of extrapulmonary and FFPE samples, when bacterial load is low, and culture is not even possible. Sensitivities of Xpert^®^ MTB/RIF and its ultra version reported in fresh non-FFPE clinical samples, so far, are always higher than 60% (Tortoli et al., 2012; Sauzullo et al., 2016; Lee et al., 2017; Bahr et al., 2018; Dorman et al., 2018; Sarfaraz et al., 2018; Sulis et al., 2018; Aydemir et al., 2019; Kohli et al., 2021) and can reach more than 90% only in some body districts (Mazzola et al., 2016). The few data available regarding the Xpert assay performances in FFPE samples are based on a few clinical biopsies and reported a wide range of sensitivities, which were not even concordant (from 97.6% in the case of the Ultra version to 53.2% in the case of the first MTB/RIF assay) (Seo et al., 2014; Du et al., 2019; Njau et al., 2019; Budvytiene and Banaei, 2020; Huang et al., 2020). Some of these reports also suggest different sensitivities against the sites of biopsies (i.e., lymph node vs. non-lymph nodes sites) (Polepole et al., 2017).

Hence, performing an accurate, quantitative, and sensitive MTB diagnosis is still a challenge. ddPCR is a third-generation PCR technology that allows an absolute quantification of nucleic acid molecules. This methodology is widely used to diagnose infectious diseases for its good accuracy, sensitivity, and specificity (Caviglia et al., 2018; Alteri et al., 2020; Chen et al., 2021; Malin et al., 2021), and has been successfully applied in different samples and clinical settings like SARS-CoV-2 (Alteri et al., 2020), HPV (Malin et al., 2021), HBV (Caviglia et al., 2018), or HIV (Strain et al., 2013), as some examples.

To the best of our knowledge, this is the first study that compared the performances of ddPCR with the MTB culture, recognized to be the gold standard for the MTB diagnosis, thanks to the availability of one fresh and one FFPE subsamples from the same biopsy.

In brief, we defined the performances of two duplex ddPCR assays targeting the multi-copy MTB gene *IS6110* or the single-copy MTB gene *rpoB*, respectively, and the human albumin as housekeeping gene. Introducing a reference gene in the MTB ddPCR assay was helpful in measuring and reducing the errors from variations among the samples, defining efficiency of DNA extraction and amplifications. The use of the reference gene was also important for normalizing and providing accurate quantification of MTB copy numbers, expressed in our study as per 10^6^ human cells.

By comparing the performances of *IS6110* and *rpoB* ddPCR assays, we confirmed that targeting a multicopy gene like *IS6110* guarantees a sensitive and reliable MTB detection (LOD 17 vs. 40 copies/10^6^ human cells) (Bahador et al., 2005; Kolia-Diafouka et al., 2019). According to the approach by Armbruster and Pry (2008), the LoB were 0 copies/reaction for both *IS6110* and *rpoB*. Probit analysis predicted a LoD of 14 copies/10^6^ cells for *IS6110* and 40 copies/10^6^ cells for *rpoB*.

This ddPCR assay was non-inferior to the reference culture method, failing to find bacilli in only one 12-day culture-positive biopsy. This biopsy resulted in MTB negative by both Xpert and AFB methods, suggesting the presence of a paucibacillary TB disease. In line with this hypothesis, the negative ddPCR result could be caused by the absence of MTB inclusions in the FFPE subsample.

Both ddPCR assays allow to detect MTB where AFB and Xpert failed. Indeed, when the rpoB and IS6110 loads detected by ddPCR were reported against AFB and Xpert results, the copies of bacilli were lower in AFB/Xpert-negative samples with respect to AFB/Xpert-positive samples, thus, highlighting the high efficiency of ddPCR assay in diagnosing also low bacterial loads.

About paucibacillary TB disease, maintaining two mycobacterial targets like *rpoB* and *IS6110* might allow to easily discriminate between high (characterized by double positive results) and low (characterized by single positive result) mycobacteria loads. This approach can also be used as a proxy measure for monitoring anti-TB treatment efficacy over time.

No difference in sensitivity and specificity of the ddPCR-based assays was found between patients with and without a history of tuberculosis, nor a difference was found against sex, age, or site of biopsies. Both assays maintain the ability to rule out MTB from culture-negative samples (specificity: 100%).

Our results are consistent with other published papers (Patterson et al., 2018; Song et al., 2018; Cho et al., 2020) reporting the rapid detection of MTB DNA in clinical or cultural samples by ddPCR system. In 2017, Yang et al. (2017) used a *IS6110* ddPCR assay to quantify MTB DNA in the whole blood of patients with pulmonary and extrapulmonary TB, proving that this technique can have the potential to be included in TB routine diagnosis. Two years later, Nyaruaba et al. (2019) stated that ddPCR technology offers enormous advantages for MTB diagnosis, such as unparalleled sensitivity, high precision, and absolute quantification, over common molecular diagnostic platforms like the qPCR. In 2020, Cao et al. (2020) used a homemade *IS6110* ddPCR assay for MTB diagnosis in FFPE samples. Recently, a single dye duplex ddPCR protocol targeting two different MTB IS demonstrates the superiority of this system with respect to qPCR in the detection of MTB in different culture isolates (Nyaruaba et al., 2020).

This preliminary study has some limitations that need to be discussed. First, the monocentric nature of the study prevented us to collect a large number of clinical samples by different body districts, limiting the possibility to draw certain statements. A multicenter study with larger sample size could be helpful and supportive in confirming the results obtained, including the sensitivity and the specificity here reported. Moreover, the Xpert method was only performed on fresh subsamples. This avoided the possibility to compare the performances of ddPCR assay and Xpert method in FFPE subsamples. Ultra-version of the Xpert^®^ MTB/RIF, developed to improve the detection of paucibacillary disease, was not available at the time of the study, and thus, its performance on both fresh and FFPE samples was not assessed. No clinical follow-up was available, and thus, no correlation between mycobacteria load and clinical outcome can be performed. In addition, the negative control population was not selected against other diseases like HIV, asthma, *Leishmania*, toxoplasma, diabetes, and neither this information was retrospectively available. Even if in this study all positive ddPCR results were confirmed to be positive by culture, these molecular assays are unable to discriminate between viable or non-viable bacilli.

Moreover, some disadvantages of ddPCR over other molecular methods need to be mentioned: (1) the system is not widely available; (2) ddPCR implementation is more complex than other standard molecular methods and needs specialized personnel; and (3) the cost per ddPCR reaction is not cheaper than other standard molecular methods (Hindson et al., 2013; Campomenosi et al., 2016).

In spite of these limitations, our study showed preliminary evidence regarding the highly sensitive, accurate, and rapid MTB diagnosis in FFPE samples by ddPCR methods, as defined by the high concordance between IS6110 assay and culture results. The quantitative approach of ddPCR and its performances independent of body districts make this system able to differentiate high bacillary load multisystemic disseminated condition from paucibacillary anatomically compartmentalized TB. Considering these results, the ddPCR approach can be safely introduced in the clinical routine to accelerate MTB diagnosis with respect to culture, as well as to provide reliable results when culture remains unavailable, like in the case of FFPE samples.

## Data Availability Statement

The original contributions presented in the study are included in the article/Supplementary Material, further inquiries can be directed to the corresponding author.

## Author Contributions

CA, MA, RS, CFP, and EM contributed to the conception and design of the study. ST, CV, FS, SMV, EB, and EM provided the samples. MA and RS designed and conducted the experiments with the help of CL, SR, VM, ST, LG, LC, and GM. CR, VC, CV, LRC, AB, and AG helped with the clinical characterization of the patients. MA, RS, and CA analyzed and interpreted the data and wrote the manuscript. CA, CFP, EM, AB, and AG critically revised the manuscript. All authors contributed to the article and approved the submitted version.

## Conflict of Interest

The authors declare that the research was conducted in the absence of any commercial or financial relationships that could be construed as a potential conflict of interest.

## Publisher’s Note

All claims expressed in this article are solely those of the authors and do not necessarily represent those of their affiliated organizations, or those of the publisher, the editors and the reviewers. Any product that may be evaluated in this article, or claim that may be made by its manufacturer, is not guaranteed or endorsed by the publisher.

## Supplementary Material

Supplementary Figure 1 Quantasoft panel for *IS6110* (green) and *rpoB* (blue) of all 20 negative samples, 2 non-tuberculous extracts [*M. abscessus* subsp. *abscessus* (MBABAB) and *M. abscessus* subsp. *bolletii* (MBABBO)], and 1 positive control.

Supplementary Figure 2 Quantasoft panel for *IS6110* (green) and *rpoB* (blue) of positive control (Pos Ctrl; H37Rv), four positive samples (repeated in duplicate), and a negative sample (repeated in duplicate).

Supplementary Figure 3 Human albumine quantification in the 89 samples, expressed as copies/μl. Dots represent MTB positive culture samples, while triangles represent MTB negative culture samples; bars represent median and interquartile range (IQR).

Supplementary Figure 4 MTB *rpoB* and *IS6110* load against new and past MTB diagnoses **(A,B)**, sex **(C,D)**, age **(E,F)**, and anatomical compartments **(G,H)**. Each value was represented by a dot; bars represent median and interquartile range (IQR). *p*-Values were calculated by Wilcoxon rank sum test and Kruskal–Wallis where necessary.
