# Supplementary material for: Rapid Detection and Quantification of Mycobacterium tuberculosis DNA in Paraffinized Samples by Droplet Digital PCR: A Preliminary Study
Source: Front Microbiol. 2021 Sep 13;12:727774. doi: 10.3389/fmicb.2021.727774 (PMC8475183; doi:10.3389/fmicb.2021.727774)
Supplement: Supplementary file 1 [file Table_1.DOCX]

| **Year** | **Number (%) of FFPE samples collected** | **Number (%) of culture MTB positive samples** |
| --- | --- | --- |
| 2013 | 8 (9.0) | 8 (100.0) |
| 2014 | 16 (18.0) | 16 (100.0) |
| 2015 | 17 (19.1) | 17 (100.0) |
| 2016 | 6 (6.7) | 6 (100.0) |
| 2017 | 8 (9.0) | 8 (100.0) |
| 2018 | 19 (21.3) | 10 (52.6) |
| 2019 | 15 (16.9) | 3 (20.0) |

**Supplementary table 1:** The distribution of FFPE samples collected each year.

FFPE: formalin-fixed and paraffin-embedded samples
