# Supplementary figures and images for: Rapid Detection and Quantification of Mycobacterium tuberculosis DNA in Paraffinized Samples by Droplet Digital PCR: A Preliminary Study
Source: Front Microbiol. 2021 Sep 13;12:727774. doi: 10.3389/fmicb.2021.727774 (PMC8475183; doi:10.3389/fmicb.2021.727774)

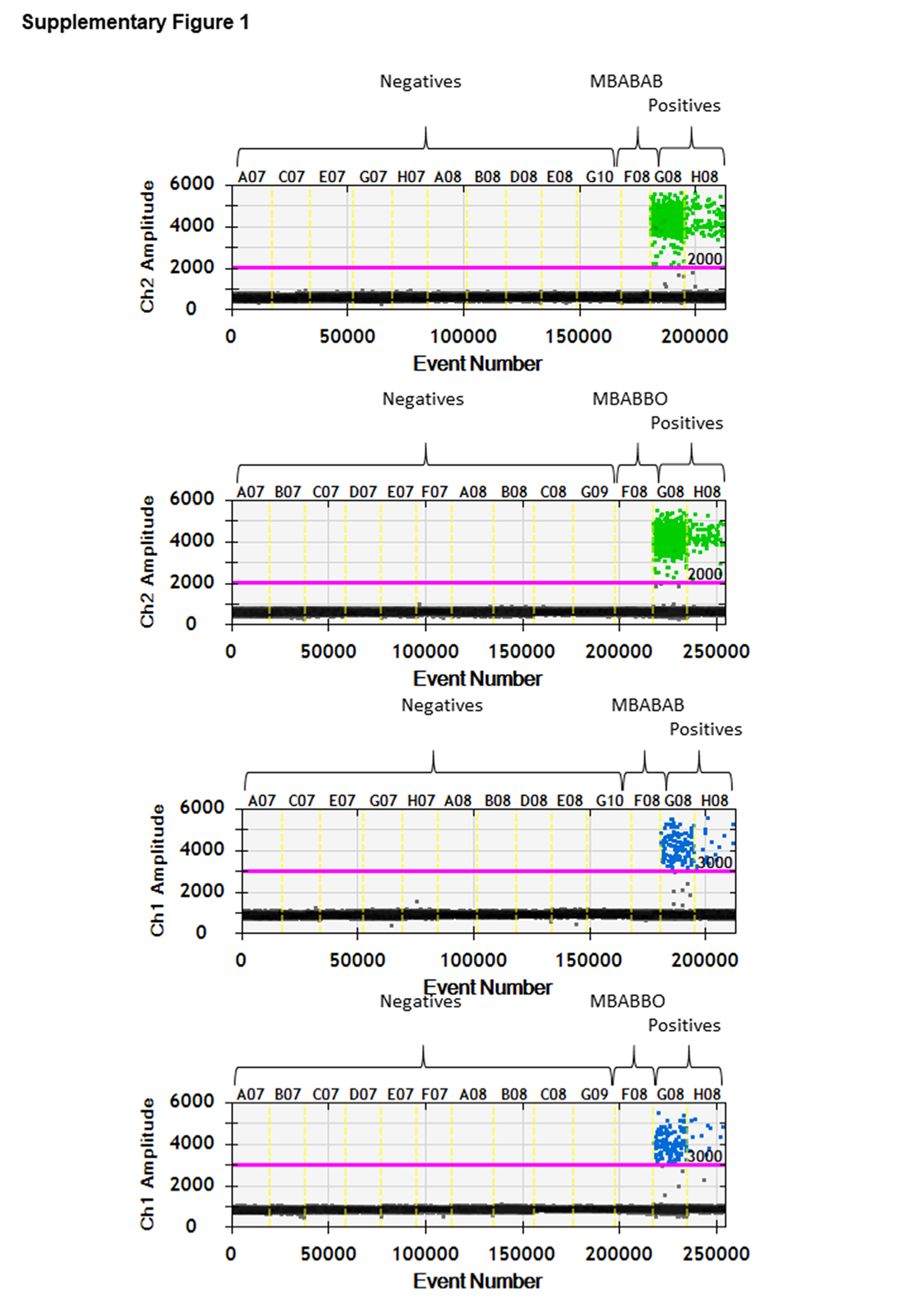

Supplement: Supplementary Figure 1 — Quantasoft panel for IS6110 (green) and rpoB (blue) of all 20 negative samples, 2 non-tuberculous extracts [M. abscessus subsp. abscessus (MBABAB) and M. abscessus subsp. bolletii (MBABBO)], and 1 positive control. [file Image_1.TIF]

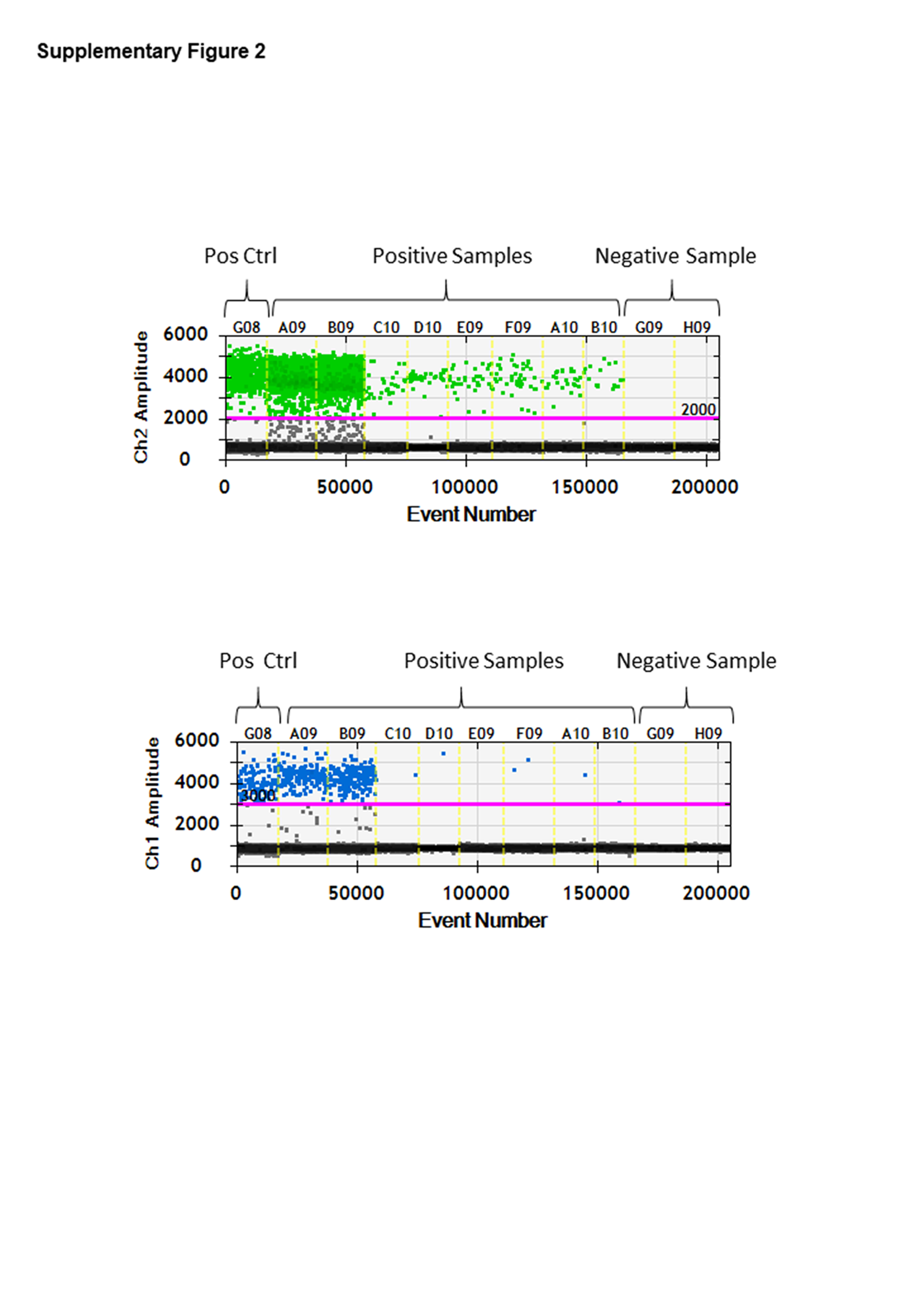

Supplement: Supplementary Figure 2 — Quantasoft panel for IS6110 (green) and rpoB (blue) of positive control (Pos Ctrl; H37Rv), four positive samples (repeated in duplicate), and a negative sample (repeated in duplicate). [file Image_2.TIF]

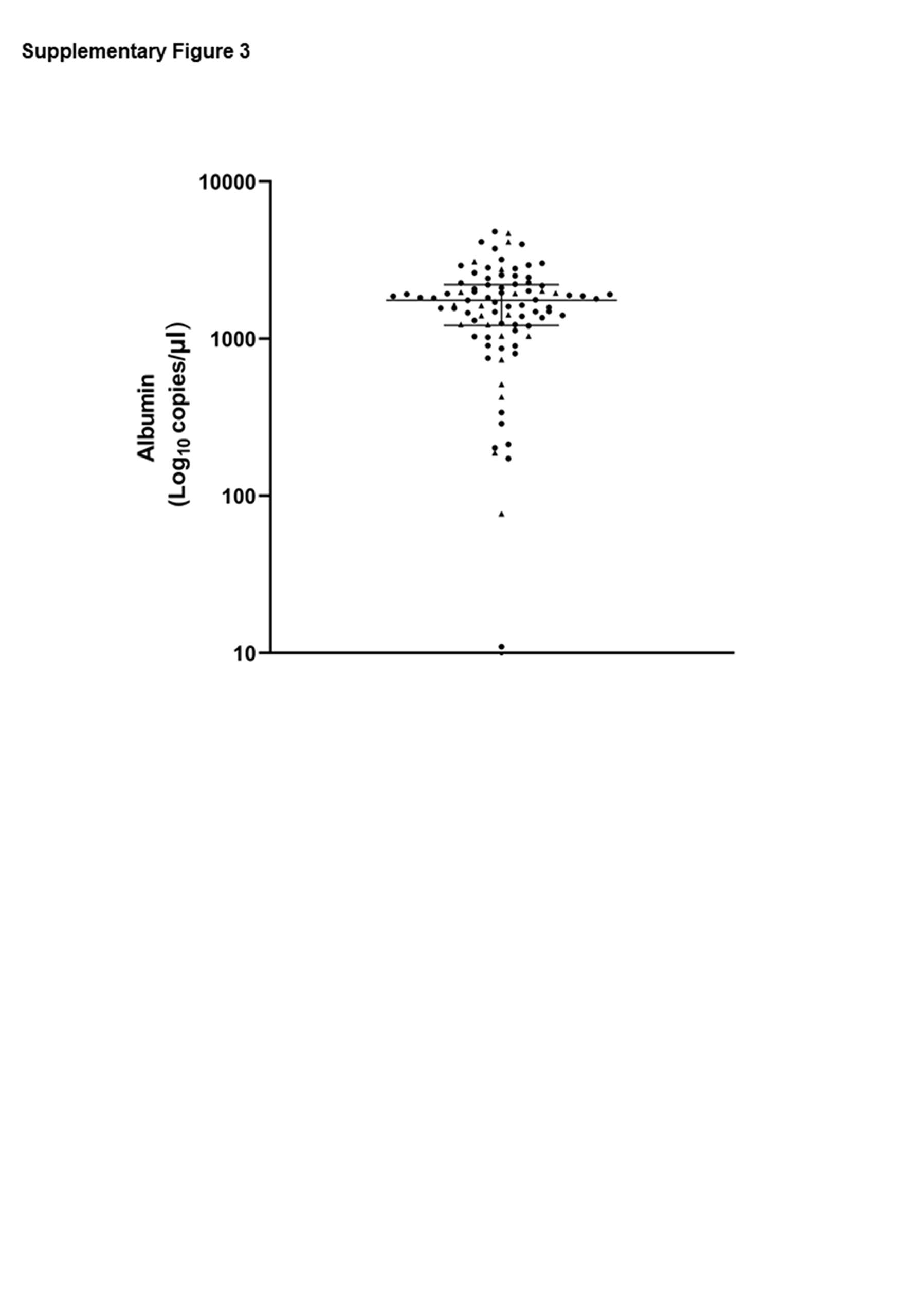

Supplement: Supplementary Figure 3 — Human albumine quantification in the 89 samples, expressed as copies/μl. Dots represent MTB positive culture samples, while triangles represent MTB negative culture samples; bars represent median and interquartile range (IQR). [file Image_3.TIF]

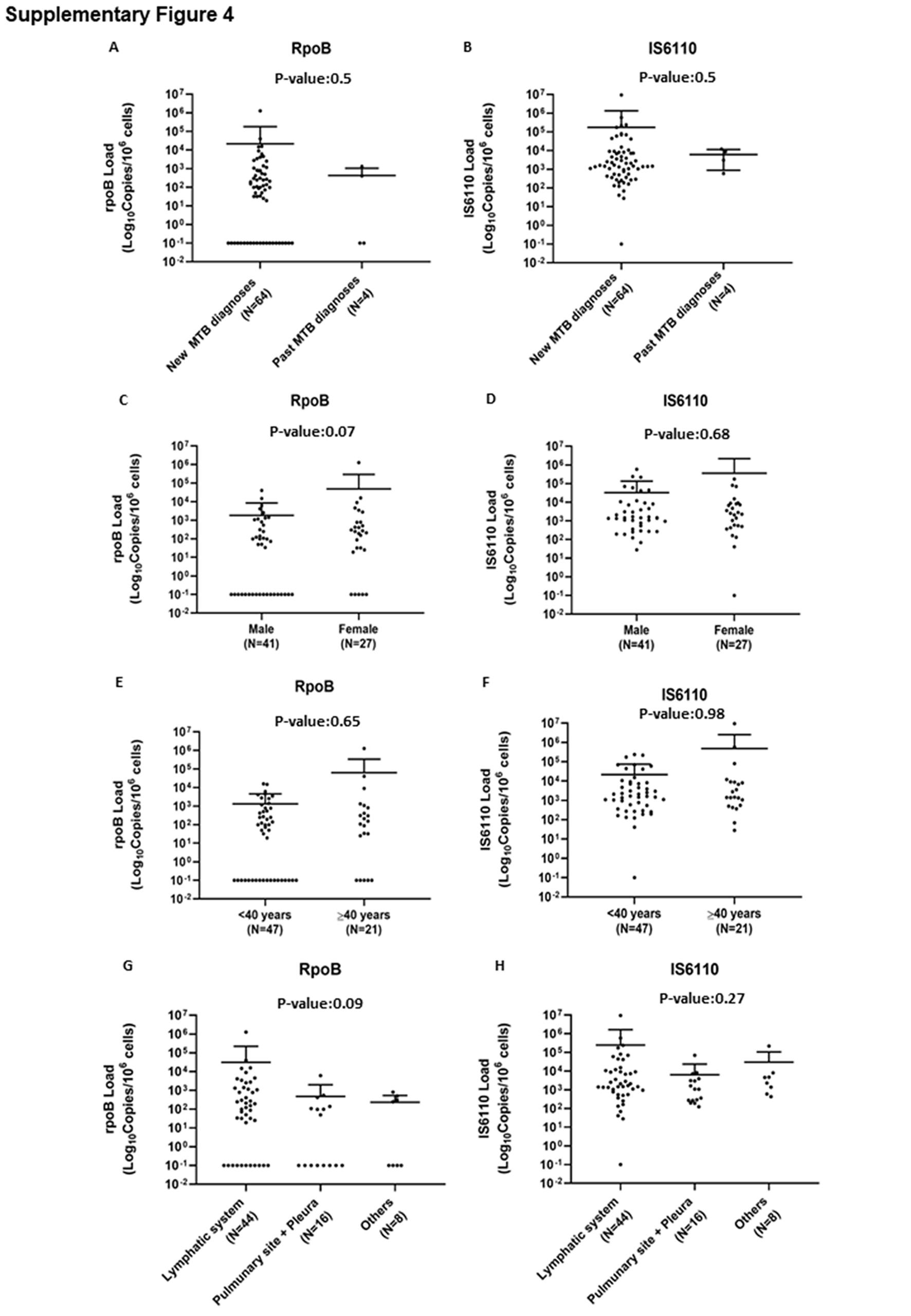

Supplement: Supplementary Figure 4 — MTB rpoB and IS6110 load against new and past MTB diagnoses (A,B), sex (C,D), age (E,F), and anatomical compartments (G,H). Each value was represented by a dot; bars represent median and interquartile range (IQR). p-Values were calculated by Wilcoxon rank sum test and Kruskal–Wallis where necessary. [file Image_4.TIF]
